# Eliciting women’s cervical screening preferences: a mixed methods systematic review protocol

**DOI:** 10.1186/s13643-016-0310-9

**Published:** 2016-08-11

**Authors:** Brianne Wood, Susan Rogers Van Katwyk, Ziad El-Khatib, Susan McFaul, Monica Taljaard, Erica Wright, Ian D. Graham, Julian Little

**Affiliations:** 1 School of Epidemiology, Public Health and Preventive Medicine, University of Ottawa, Roger-Guindon Hall, Room 3105, 451 Smyth Road, Ottawa, K1H 8M5 Ontario Canada; 2 Department of Public Health Sciences, K9, Karolinska Institutet, Tomtebodavägen 18A; Widerströmska huset, 171 77 Stockholm, Sweden; 3 World Health Programme, Université du Québec en Abitibi-Témiscamingue, 79, rue Côté Notre-Dame-du-Nord, Québec, J0Z 3B0 Canada; 4 Department of Obstetrics and Gynecology, University of Ottawa, 1919 Riverside Dr., Suite 201, Ottawa, K1H 1A2 Ontario Canada; 5 Clinical Epidemiology Program, Ottawa Hospital Research Institute, 725 Parkdale Ave., Ottawa, K1Y 4E9 Ontario Canada; 6 Health Sciences Library, University of Ottawa, Roger-Guindon Hall, Room 1020, 451 Smyth Road, Ottawa, K1H 8M5 Ontario Canada

## Abstract

**Background:**

With the accumulation of evidence regarding potential harms of cancer screening in recent years, researchers, policy-makers, and the public are becoming more critical of population-based cancer screening. Consequently, a high-quality cancer screening program should consider individuals’ values and preferences when determining recommendations. In cervical cancer screening, offering women autonomy is considered a “person-centered” approach to health care services; however, it may impact the effectiveness of the program should women choose to not participate. As part of a larger project to investigate women’s cervical screening preferences and correlates of these preferences, this systematic review will capture quantitative and qualitative investigations of women’s cervical screening preferences and the methods used to elicit them.

**Design and methods:**

This mixed methods synthesis will use a thematic analysis approach to synthesize qualitative, quantitative, and mixed methods evidence. This protocol describes the methods that will be used in this investigation. A search strategy has been developed with a health librarian and peer reviewed using PRESS. Based on this strategy, five databases and the gray literature will be searched for studies that meet the inclusion criteria. The quality of the included individual studies will be examined using the Mixed Methods Appraisal Tool. Three reviewers will extract data from the primary studies on the tools or instruments used to elicit women’s preferences regarding cervical cancer screening, theoretical frameworks used, outcomes measured, the outstanding themes from quantitative and qualitative evidence, and the identified preferences for cervical cancer screening. We will describe the relationships between study results and the study population, “intervention” (e.g., tool or instrument), and context. We will follow the PRISMA reporting guideline. We will compare findings across studies and between study methods (e.g., qualitative versus quantitative study designs). The strength of the synthesized findings will be assessed using the validated GRADE and CERQual tool.

**Discussion:**

This review will inform the development of a tool to elicit women’s cervical screening preferences. Understanding the methods used to elicit women’s preferences and what is known about women’s cervical screening preferences will be useful for guideline developers who wish to incorporate a woman-centered approach specifically for cervical screening guidelines.

**Systematic review registration:**

PROSPERO CRD42016035737

**Electronic supplementary material:**

The online version of this article (doi:10.1186/s13643-016-0310-9) contains supplementary material, which is available to authorized users.

## Background

Health care providers and public health organizations have enthusiastically promoted the importance of cancer screening for several years as some cancer screening programs have been found to reduce mortality from the disease [1, 2]. However, debates about the risks and benefits of cancer screening are occurring in the academic and the public domains [3–5]. For example, several organizations do not recommend population-based prostate-specific antigen testing because the harms outweigh the benefits [6–8], and recent publications question the optimal age range for mammography screening [9, 10]. In some jurisdictions, people are offered choices about what method of colorectal cancer screening they would prefer, though access to these options may be variable [11–14]. While most health professionals agree that personalized cancer screening would benefit their patients, the decision-making process is usually left to clinicians. Often, patients are not presented with a decision between screening options for pragmatic reasons: health systems are organized to evaluate and reimburse clinicians based on specific guideline adherence (which may not include a personalized pathway), clinicians may disagree with personalized recommendations, or certain options may require extra resources [15]. Additionally, health professionals have expressed concern that options may deter individuals from pursuing cancer screening, which could affect cancer rates among the population, although there appears to be no evidence to substantiate this [13, 15, 16] The benefits of early cancer detection and potential treatment must be weighed against harms such as complications from the screening process (e.g., perforation from colonoscopy), potential for false positives and false negatives, anxiety resulting from receiving abnormal results, and treatment of conditions that may not have developed into a problem, which can vary widely based on a patient’s age and health status [15].

Cervical cancer screening has avoided some of the media controversy that has affected breast, prostate, and recently colorectal screening programs because the Papanicolaou (Pap) test has successfully reduced the incidence of invasive cervical cancer and associated mortality [17–19]. In Ontario, Canada, the provincial cancer agency now recommends a new method of screening—human papillomavirus (HPV) testing—because of its increased sensitivity for detecting cervical abnormalities and high negative predictive value for high-grade cervical intra-epithelial neoplasia, compared with Pap testing [20]. Such a change in screening test would require cervical screening stakeholders to make decisions about the age range during which screening should be offered, options for sample collection methods, options for the laboratory testing, screening frequency, and recommended follow-up algorithm. In particular, HPV testing has the option for self-collection, which would allow women to take their own samples without the supervision of a health care professional, though possibly at a cost of slightly decreased diagnostic performance [21, 22]. This strategy could have implications for access to screening, screening frequency, follow-up algorithms, and how screening results are communicated to women. As for most other health care decisions, women and health care professionals would be expected to make cervical screening choices that maximize the potential for early detection of a disease and minimize harm [23]. Population-level benefits and harms are balanced on a broader scale, looking at reduced disease-specific morbidity and mortality compared to overly intensive screening strategies [24]. Cancer screening decisions such as this one are inherently preference sensitive at both the individual level and program level, as decision-makers are required to understand how they value the benefits and harms for the different elements of the decision [25–27]. Therefore, individuals can identify their preferred choice by considering how they value each option’s benefits and risks.

According to the Ottawa Decision Support Framework (ODSF), *preference* refers to the most appealing option of a decision for an individual, which is identified through *values clarification* [28, 29]. Values clarification is a process in which individuals identify the characteristics that matter most to them for a specific health decision. In cancer screening, individuals would compare and contrast what the tests measure, their diagnostic performance, how the tests are performed, follow-up procedures, and the potential consequences of participating in screening, and, subsequently, identify a plan to act on these preferences [30]. In the ODSF, values clarification and preference elicitation are part of shared decision-making processes, in which individuals are supported in these exercises by a decision support person [29]. Llewellyn-Thomas and Crump identified interactive and non-interactive, fine-grained, and coarse-grained approaches to values clarification and preference elicitation for health care decision-making in their 2013 review [29]. As the authors note, there are conceptual and methodological issues between the different values clarification/preference eliciting approaches, and further research is required to determine the appropriate approach based on different subgroups of patients. Considering the evolving and complex evidence in cervical cancer screening, it is imperative that women are supported in these preference-sensitive health care decisions, for individual-level decision-making and through policies that reflect women’s preferences.

Systematic reviews have shown that more women may participate in cervical screening if they can self-collect their samples [31, 32]. Other reviews have indicated that women tend to prefer active follow-up of abnormal screening results, predominantly referral to colposcopy, to observation, and predominantly repeated Pap tests [33]. In studies investigating women’s views on HPV testing as part of cervical cancer screening [34], some respondents had trouble interpreting information about cervical cancer screening [33]. This was related to psychosocial burden of positive results, acceptability of the testing process, and women’s informational needs [34]. There is weak evidence that women’s participation in cancer screening is related to the presentation of the screening information as well as emotional and cognitive responses to the invitation to be screened [35]. While there is a growing body of evidence focusing on women’s attitudes and beliefs in cervical screening, it is unclear whether these studies consider women’s preferences in terms of making informed decisions, which undermines the potential for improved screening for women. To the best of our knowledge, no systematic review has described or compared methods for eliciting women’s cervical screening preferences and the outcomes of these approaches. The proposed review will contribute to an assessment of women’s needs in making decisions about primary cervical cancer screening and inform policy-makers and guideline developers about these needs. These findings will be used in an investigation of women’s cervical screening preferences in Ontario, Canada.

### Aim

The objectives of this systematic review are

1. to synthesize and describe what is known about methods used to elicit women’s preferences in primary cervical cancer screening;
2. to summarize the evidence on women’s preferences for cervical cancer screening.

## Methods

This protocol has been prepared following Preferred Reporting Items for Systematic Review and Meta-Analysis Protocols (PRISMA-P) guidelines for systematic review protocols [36]. The PRISMA-P checklist is included as Additional file 1. The review protocol has been registered with PROSPERO (CRD42016035737).

### Conceptual framework

This systematic review is part of an assessment of women’s needs in identifying their preferences and making decisions about cervical cancer screening, taking into account issues laid out in the ODSF [28, 37]. We will apply this framework to conduct a thematic analysis of the synthesis results, looking at determinants of preferences and decision-making, methods used to support decision-making or elicit preferences, and short- and long-term outcomes [28, 29, 37]. In addition to summarizing different techniques of eliciting preferences, we will compare theoretical frameworks used to elicit preferences, processes used to design these techniques, outcomes including construct validity, clinical outcomes, and decision quality, the identified preferences for cervical cancer screening, and how these preferences are influenced by context.

### Eligibility

We will evaluate qualitative and quantitative studies that describe and/or examine methods used to elicit women’s cervical screening preferences. These studies will involve women being presented with multiple options for primary cervical cancer screening and identifying the most favored option [29]. We will include studies of women who are presented with options at the point of decision-making, as well as women presented with a hypothetical decision. The criteria for inclusion in this review are summarized in Table 1. For all study designs, we will examine studies in which women are 18 years of age or older and are eligible for cervical cancer screening. We will include articles that are based on direct enquiry with eligible women with no restriction on publication dates or publication status. For articles not published in English that the study team is unable to translate, we will attempt to contact the study authors to see if there are any English translations available. All study designs will be included. Mixed methods studies that satisfy corresponding inclusion and exclusion criteria will also be included (eligibility will be determined separately for the quantitative and qualitative components). We will examine each of the primary studies from any systematic review or meta-analysis for individual inclusion into the review. Opinion pieces will not be included.

**Table 1:** Eligibility criteria for studies in systematic review

Inclusion criteria	Exclusion criteria
- Participants: women aged ≥18 years - Assessment of women’s cervical screening preferences, either current or hypothetical - Investigation of decision-making needs of women who are considering cervical cancer screening - Both qualitative and quantitative study designs	- Interventions targeting health care providers, policy-makers (e.g., program decision-makers), or technical aspects of cervical cancer screening or treatment - Reports of interventions that aim to increase cervical screening uptake in which the content of the intervention was not described or evaluated - Studies reporting predictors of cervical screening uptake that are not related to a decision-making process - Opinion pieces - Studies that do not adequately report the study design, methods, and interpretation of the research - Studies that do not clearly demonstrate ethical conduct or ethical reporting of research

### Information sources

We will search MEDLINE, Embase, PsycINFO, CINAHL, and Cochrane Library databases, for English articles from inception to March 2016. The gray literature will be searched by scanning ProQuest Dissertations and Theses, relevant Canadian health research/policy databases, Canadian health information networks, and known relevant Canadian conference proceedings. Our gray literature search is limited to Canadian resources as the findings from this review will inform an investigation of cervical screening preferences in the Canadian context. A search strategy and its translations to other databases were developed in collaboration with a health sciences librarian at the University of Ottawa and peer reviewed using PRESS [38]. These strategies combined free-text words and subject headings, derived from the following broad topic areas: cervical cancer, early detection of cervical cancer, and patient preferences. The MEDLINE search strategy is appended (Additional file 2). We will search citations of the included studies using SCOPUS to supplement our search of the main bibliographic databases. Reference lists of articles that were selected for full-text review will be scanned for additional relevant material. Subject matter specialists on the review team will be asked to identify further resources for inclusion.

### Study identification and data extraction

Two of three reviewers will independently review the studies in three stages: (i) title and abstract screening for inclusion; (ii) full-text screening for inclusion; and (iii) data extraction for eligible studies. All studies which initially appear to meet the inclusion criteria but on inspection of the full-text paper are not eligible will be detailed in a table. We will identify single studies that are reported in multiple publications by juxtaposing the author lists and comparing sample size and setting characteristics. Multiple reports of the same study will be considered for inclusion, but the data will be extracted for single studies from the report deemed the most relevant by the two reviewers. Variation in results reported in multiple publications will be noted as limitations in the appraisal of each study. Characteristics of excluded studies’ together with reasons for their exclusion will be recorded. Disagreements regarding eligibility of studies will be resolved by the third reviewer.

Data will be extracted independently by two of the three reviewers, according to the list in Table 2. Mendeley (www.mendeley.com) and Covidence (www.covidence.org) will be used for citation management, screening, and data extraction. Data will be extracted to preserve the context of the findings, as described by Sandelowski et al. [39] using pre-defined data extraction forms. Descriptive themes of preference eliciting methods and preferred cervical screening options will be developed for each primary study based on the text-in-context results [39]. Differences will be resolved through discussion between the two reviewers, and if necessary, through final consensus with a third reviewer. Customized data extraction sheets will be pilot tested by all reviewers prior to the data extraction phase.

**Table 2:** Data extraction fields for synthesis

Study details	Eligibility	Study design	Clinical/decision-making issues	Summary
Author, year Reference ID Publication type (i.e., full report or abstract) Country of corresponding author Sources of funding Ethical considerations (approval from committee, informed consent, positive risk/benefit assessment)	Direct enquiry with women considering screening (18+ years) Preferences elicited (options presented, women indicate most favored option)	Methodological characteristics/epidemiological design Population Setting/context Time period Method of recruitment Method of data collection Method of data analysis Theoretical frameworks Concept definitions	Method/procedure of preference elicitation (and/or values clarification) Design process of preference elicitation/values clarification technique(s) Comparators Results (preferred option, strength of preferred option, evaluation of preference eliciting technique) Total number of participants Objective 1: magnitude and significance; study-specific conceptions Objective 2: magnitude and significance; study-specific conceptions	Authors’ overall conclusions Quality assessment Reviewer’s comments

### Outcomes

Objective 1: The first objective is to synthesize what is known about methods used to elicit women’s preferences in primary cervical cancer screening, including

- Approaches used to elicit women’s preferences for screening technologies (e.g., interactive versus non-interactive, coarse-grained versus fine-grained, other);
- Methods used to evaluate the appropriateness of women’s preferences (e.g., strength of preferred option, change in decisional conflict, acceptability of option’s characteristics, change in knowledge, attitudes, or behavior);
- Reported indicators of effectiveness of methods to elicit preferences (e.g., observed strengths of preferences, observed changes in decisional conflict, observed acceptability of options, observed changes in knowledge, attitudes, or behavior);
- Advantages and disadvantages of the different methods to elicit preferences.

Objective 2: The second objective is to summarize the evidence on women’s preferences for cervical cancer screening, including

- List of options in cervical screening decisions for women;
- Women’s preferences for cervical screening (by proportions), by jurisdiction;
- Description of important values for making cervical screening decisions.

### Quality assessment/risk of bias

The Mixed Methods Appraisal Tool (MMAT) [40, 41] will be used to appraise the qualitative, quantitative, and mixed methods studies for this review. Two reviewers will independently appraise relevant studies, and disagreement will be resolved through consensus, in discussion with a third reviewer. Studies will be included in the final review if they score 50 % or greater, according to the scoring scheme [40].

The Grading of Recommendations, Assessment, Development and Evaluation (GRADE) guidelines will be used to present a summary of findings and to qualify the strength of individual findings for the quantitative and qualitative syntheses separately, using GRADE profiler and the Confidence in the Evidence from Reviews of Qualitative Evidence (CERQual) tool, respectively [42–45].

### Analysis

The quantitative and qualitative evidence will be analyzed separately and then combined in the final stage of analysis, similar to Goldsmith’s integrated methodology [46]. We expect considerable heterogeneity between studies and therefore that a statistical meta-analysis would not be appropriate. A tabular summary will be created to describe all of the characteristics of the included studies, as well as key preference eliciting methodologies [47], the advantages and disadvantages of their approaches, and their conclusions regarding women’s preferences based on the text-in-context analysis.

Objective 1: We will produce a quantitative evidence table that describes the main types of preference eliciting methods identified in the quantitative body of literature and may also contain relevant evaluative measurements of the different techniques. Methods will be categorized using themes from a textual analysis technique when reviewing the qualitative literature, using the classification as outlined by Llewellyn-Thomas and Crump [29] as an initial reference. A similar evidence summary table of the included qualitative studies will be developed, conveying study characteristics, context, and outstanding themes. In both of these summary thematic tables, we will also include a column that indicates if and how the themes map onto the ODSF concepts.

Objective 2: Women’s preferences will be organized into themes using the text-in-context analysis and presented in a table, alongside the options available and values that women identified as important for their decision-making. Quantitative evidence will be synthesized and presented separately from the qualitative evidence.

For both objectives, summary of findings tables will be developed according to the GRADE guidelines (i.e., GRADE and CERQual) for syntheses of the quantitative and qualitative evidence separately. Evidence profiles [46] will be created for each finding to determine recommendations for eliciting women’s cervical screening preferences, for each objective. These profiles will be presented in a table that denotes which studies contribute evidence toward a particular theme, the associated type of evidence (i.e., qualitative or quantitative), related quality appraisal scores from the MMAT, the consistency of the finding across the studies (e.g., noting any contradictions or missingness), the applicability of the evidence to the Ontario/Canadian population, and any other factors that might influence the usability of the evidence. Each evidence profile will include overall assessments from the GRADE and CERQual summary of findings tables. Using the categories defined by Goldsmith et al. [46], analytic themes will be graded as “definite” or “suggestive,” based on the GRADE and CERQual assessments. Items which were classified as “high” or “moderate” according to GRADE or CERQual will be given a “definite” recommendation, and items classified as “low” and/or “very low” will be denoted “suggestive.” This method of synthesis will clarify the important dimensions of eliciting women’s preferences for cervical screening, giving equal value to quantitative and qualitative evidence. “Definite” recommendations from the evidence profile will be used to develop a tool to elicit women’s screening preferences in practice. “Suggestive” recommendations will be discussed with local stakeholders to determine their relevance for this particular project and can be used to guide further research on women’s screening preferences.

## Discussion

This study will be the first systematic review synthesizing methods used to elicit preferences for cervical cancer screening. This review constitutes the first part of a needs assessment for the development of a tool to elicit women’s preferences for different cervical cancer screening options. The findings of this review will inform a qualitative investigation of women’s cervical screening decision-making in Canada.

### Strengths and limitations

Recommendations for mixed methods systematic reviews were developed only recently [46, 48–50], and therefore, best practices have not yet been fully established. While this study will use some validated approaches to synthesizing quantitative and qualitative evidence, it may be challenging to apply the results of this synthesis because of the heterogeneity of the evidence. This mixed methods approach values quantitative and qualitative evidence equally, offering a more complex understanding of how women make decisions about cervical cancer screening.

As with any knowledge synthesis, this review will be subject to particular biases, including publication bias and language bias. This review will be examining the gray literature to attempt to find relevant unpublished work. If there are relevant articles identified that are not published in English, the authors will seek to translate the document internally or will attempt to retrieve a translation by contacting the author. We expect that the heterogeneity in definitions of “preference” and potential reporting biases may complicate the synthesis of across a diverse body of literature. This review will contribute to improved understanding of how best to elicit cancer screening preferences from women, which women, health care providers, and policy-makers may value. A thematic synthesis may not provide the same certainty as a meta-analysis, though an evidence table with descriptive themes is important for comparing quantitative and qualitative evidence and will be useful for generating recommendations from the final aggregation.

This knowledge synthesis will summarize and compare the content and methodologies used to elicit women’s preferences of cervical screening. A pooled synthesis will highlight what is known about methods used to elicit women’s preferences in cervical screening and their identified preferences across a diverse body of evidence. This information will contribute to the design of a tool to elicit women’s preferences for cervical screening for potential use by health professionals in developing woman-centered cervical screening recommendations. These findings will also suggest gaps in the knowledge of women’s cervical screening preferences that can be addressed in future research.

## Abbreviations

CERQual, Confidence in the Evidence from Reviews of Qualitative Evidence; GRADE, Grading of Recommendations, Assessment, Development and Evaluation; HPV, human papillomavirus; MMAT, mixed methods appraisal tool; ODSF, Ottawa Decision Support Framework; Pap, Papanicolaou

## Additional files

Additional file 1: PRISMA-P checklist. (PDF 219 kb) Additional file 2: Search strategy for MEDLINE. (PDF 100 kb)
