# Supplementary material for: Eliciting women’s cervical screening preferences: a mixed methods systematic review protocol
Source: Syst Rev. 2016 Aug 11;5:136. doi: 10.1186/s13643-016-0310-9 (PMC4982264; doi:10.1186/s13643-016-0310-9)
Supplement: Additional file 2: — Search strategy for MEDLINE. (PDF 100 kb) [file 13643_2016_310_MOESM2_ESM.pdf]

Searches	Results
1	exp Uterine Cervical Neoplasms/ or exp Cervical Intraepithelial Neoplasia/
2	Papillomavirus Infections/
3	(human papilloma* or HPV).ti,ab.
4	(cerv* adj4 (cancer* or tumo?r* or neoplas* or malignan* or dysplas*)).ti,ab.
5	or/1-4
6	Mass Screening/ or "Early detection of cancer"/
7	(screen* or detect* or cytology*).ti,ab.
8	(vagina* adj2 smear*).ti,ab.
9	Self-Examination/
10	(self-exam* or self-test* or self-sampl* or self-collect*).ti,ab.
11	"Diagnostic Techniques and Procedures"/
12	Cytodiagnosis/
13	Human Papillomavirus DNA Tests/
14	(pap* adj2 (smear* or test*)).ti,ab.
15	(cerv* adj2 (smear* or screen* or cyto*)).ti,ab.
16	((human papilloma* or HPV) adj2 test*).ti,ab.
17	or/6-16
18	Patient Preference/
19	Perception/
20	decision making/ or choice behaviour/
21	Attitude to Health/ or Attitude/
22	Health Knowledge, Attitudes, Practice/
23	Motivation/
24	"patient acceptance of health care"/ or patient satisfaction/
25	(mak* adj2 decision*).ti,ab.
26	((patient* or wom?n* or girl*) adj5 (prefer* or accept* or attitude* or opinion*)).ti,ab.

27	((patient* or wom?n* or girl*) adj5 (motivat* or perception* or perspective*)).ti,ab.
28	((patient* or wom?n* or girl*) adj3 needs).ti,ab.
29	Patient-Centered Care/
30	(patient-centered or patient-centred).ti,ab.
31	or/18-30
**32**	**5 and 17 and 31**
